# Interleukin 20 regulates dendritic cell migration and expression of co-stimulatory molecules

**DOI:** 10.1186/s40591-016-0046-x

**Published:** 2016-01-26

**Authors:** Rikke Bech, Babak Jalilian, Ralf Agger, Lars Iversen, Mogens Erlandsen, Kristian Otkjaer, Claus Johansen, Søren R. Paludan, Carina A. Rosenberg, Knud Kragballe, Thomas Vorup-Jensen

**Affiliations:** Department of Dermatology, Aarhus University Hospital, Aarhus, Denmark; Department of Biomedicine, Aarhus University, Aarhus, Denmark; Department of Health Science and Technology, Aalborg University, Aalborg, Denmark; Department of Public Health - Biostatistics, Aarhus University, Aarhus, Denmark; Interdisiplinary Nanoscience Center, Aarhus University, Aarhus, Denmark; Biophysical Immunology Laboratory, Department of Biomedicine, Aarhus University, The Bartholin Building (1240), Wilhelm Meyers Allé 4, DK-8000 Aarhus C, Denmark

**Keywords:** Dendritic cells, Psoriasis, Interleukin (IL)-20, CD18 integrins, Cell migration

## Abstract

**Background:**

Psoriasis is an inflammatory disease characterized by leukocyte skin infiltration. Interestingly, recent works suggest that the migration of dendritic cells (DCs) is abnormal in psoriatic skin. DCs have significant role in regulating the function of T lymphocytes, at least in part influenced by the local environment of cytokines. In psoriatic skin lesions the expression of IL-20 is highly up-regulated. It is unclear if this cytokine has any influence on DCs.

**Methods:**

Here, we investigated the influence of IL-20 in monocyte-derived dendritic cell (MDDCs) in vitro. This work addressed IL-20 effects on DC maturation, receptor expression and signaling. By use of extra cellular matrix components mimicking the skin environment, we also studied the functional effects of IL-20 on the chemotactic migration of DCs. Based on the recent finding that CD18 integrin are shed during migration of myeloid leukocytes, the concentration of these adhesion molecules was measured in MDDCs culture supernatants post migration.

**Results:**

Following stimulation with IL-20, immature human MDDCs enhanced the expression of the co-stimulatory molecule CD86, further enabling activation of the p38 MAPK, but not the STAT3, pathway. IL-20 increased the migration of MDDCs in a biphasic response narrowly controlled by the interleukin concentration. A concomitant change in the shedding of CD18 integrins suggested that these adhesion molecules play a role in the migration of the MDDCs through the extracellular matrix layer.

**Conclusion:**

Taken together, our findings points to a possible, yet subtle, role of IL-20 in DCs migration. The biphasic response suggests that the aberrant IL-20 expression in psoriasis impedes DC migration, which could be a part of the processes that precipitates the dysregulated inflammatory response associated with this disease.

**Electronic supplementary material:**

The online version of this article (doi:10.1186/s40591-016-0046-x) contains supplementary material, which is available to authorized users.

## Background

IL-20 belongs to the IL-10 family of λ interleukins with 15–39 % identical primary structure in pairwise comparisons [1, 2]. IL-10 interleukins contribute to a great variety of cellular processes, which regulate the immune system, including antiviral activity, antibacterial proteins secretion, cell-growth stimulation, acute phase response, wound healing, anti-tumor activity, as well as induction of apoptosis [1, 2]. However, studies connecting the function of IL-20 to specific leukocyte subsets are still lacking. Studies showed that IL-20 expression in humans is associated with chronic inflammation disorders such as psoriasis and rheumatoid arthritis [3]. Furthermore, the over expression of IL-20 in transgenic mice generated psoriasis-like skin lesions [4] and treatment of human skin xenografts with IL-20 induced psoriasis-like skin lesions [5].

In psoriasis, epidermal keratinocytes are a major source of IL-20 synthesis and the transcriptional level of IL-20 is strongly upregulated in psoriatic skin lesions [5]. Other studies suggest that monocytes and maturing DCs are additional sources of IL-20 [2, 6] although more recent work also suggest that papillary mononuclear cells possibly may take up IL-20 released into the epidermis by keratinocytes [7]. DCs expressing CD40, CD86, and HLA-DR are abundant in psoriatic skin, while resolution of psoriatic lesions results in reduction of DC infiltration [8]. Normal skin contains an abundant subset of CD1a + CD207 + immature DCs, also referred to as Langerhans cells (LC). Appropriate uptake of antigen prompts the migration of LC from epidermis to the dermis and draining lymph nodes through the basement membrane. Cumberbatch et al. reported that the mobilization of immature DCs following IL-1β and TNFα stimulation was impaired in non-lesional psoriatic skin compared to the non-inflamed skin where these cytokines support the DCs migration [9]. Intriguingly, it was recently demonstrated that these findings were not due to a defect in DCs function [10] pointing to the cytokine milieu as a factor in the migrational impairment. However, there has been a paucity of studies at the cellular level to more precisely investigate these factors.

Integrins are heterodimeric cell adhesion molecules, composed of one alpha and one beta chain, which support cell-cell and cell-extracellular matrix (ECM) adhesion [11]. The family of β2 integrins (CD18) includes CD11a/CD18 (LFA-1), CD11b/CD18 (Mac-1 or complement receptor 3), and CD11c/CD18 (p150,95 or complement receptor 4) with an expression restricted to leukocytes. The role of CD11a/CD18 in psoriasis is well established from the clinical application of Efalizumab [12], which blocks the function of CD11a/CD18 and hence decreasing the influx of DCs into psoriatic skin [8]. Recently, it has been shown that the plasma concentration of shed CD18 integrins is lower in arthritis patient samples compared with healthy controls. By contrast, the concentration in synovial fluid from the inflamed arthritis patients is elevated compared with the less inflamed osteoarthritis patients [13, 14]. This points to shedding of CD18 in the zone of inflammation supporting the hypothesis of a link between cellular migration and shedding of CD18 [15]. A few studies have implicated CD18 shedding as part of chemotaxis and exposure to stress shear in neutrophils [16, 17], but experimental studies comparing migration and the shedding of CD18 in leukocytes central to adaptive immunity have not been reported earlier.

In the present study we established culturing conditions to enable the differentiation of human peripheral monocytes into DCs with an immature phenotype. We investigated the influence of IL-20 on the maturation of these monocyte-derived DCs (MDDC)s, interleukin receptor expression and signaling, as well as the function of MDDCs in cell migration and shedding of adhesion receptors. We report that IL-20 influences the expression of co-stimulatory molecules. The MDDCs express both IL-20RA and IL-20RB, which permits signaling involved in phosphorylation of p38. We found that IL-20 influenced the migration of MDDCs. These findings readily suggest a link between the function of IL-20 and trapping of DCs in psoriatic skin.

## Methods

### Isolation of monocytes from human blood

Blood samples were collected from healthy human donors according to protocols for obtaining samples from humans approved by the institutional ethical review committee. Monocytes were, then, isolated using Ficoll Paque PLUS (GE Healthcare, Little Chalfont, UK) and “Monocyte Negative Isolation Kit”™ (Gibco-Life, Waltham, MA) according to the instructions by the manufacturer. The monocytes were resuspended in RPMI-1640 culture medium containing 20mg/ml gentamicin, 2 mM l-glutamine, and 2 % (v/v) heat-inactivated human serum (Gibco-Life).

### Differentiation of monocytes into MDDCs

**Fig. 1:**
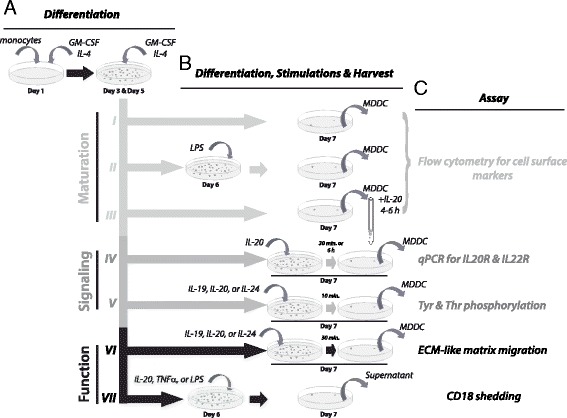
Schematic overview of the MDDCs cultures. **a** All cultures were initiated by 5 days of treatment of monocytes with GM-CSF and IL-4. During culture the medium and cytokines were replenished as indicated. **b** Separate incubations were applied for analyzing the influence of IL-20 and other stimulants on maturation (I-III), signaling (IV-V), or function (VI-VII). **c** The influence of the stimulants was tested with cellular-based assays

Isolated monocytes were cultured for a total of seven days in 6-cm Petri dishes. (Nunclon Surface™, 150288; Nunc, Kamstrup, Denmark). Cultures were kept in supplemented RPMI-1640 culture medium (described above) and incubated at 37 °C in an atmosphere with 5 % (v/v) CO_2_ and 80 % relative humidity (Fig. 1). The differentiation was initiated on Day 1 (Fig. 1a) by 20 ng/ml recombinant IL-4 (PeproTech EC, London, UK) and 100 ng/ml GM-CSF (Leukine Sargramostim™, Berlex, Richmond, CA). On Day 3 the cultures received 1.05 ml of culture medium containing 67 ng/ml IL-4 and 300 ng/ml GM-CSF and on Day 5 a volume of 4.55 ml culture medium containing 40 ng/ml IL-4 and 200 ng/ml GM-CSF was added. Following additional cultivation for two days (Incubation I, Fig. 1b) the cells were harvested by flushing the Petri dishes with ice-chilled culture medium and analyzed by flow cytometry as described below. As a positive control on the expression of maturation markers, cells were also stimulated (Fig. 1b) with 10 ng/ml of LPS (Sigma-Aldrich, St. Louis, MO) on Day 6. To investigate the role of IL-20 in maturation of DCs, cells were incubated for 4-6 h with recombinant human IL-20 [18] (Batch 022 KMK, Novo Nordisk, Måløv, Denmark) at concentrations of 0.0001-5000 ng/ml (Fig. 1b).

### Characterization of MDDCs surface marker expression

Analysis of the MDDCs expression of cell surface markers was carried out by flow cytometry for which antibodies labelled with surface markers using antibodies to HLA-DR (BD Bioscience, Franklin Lakes, NJ), CD1a (BD Biosciences), CD1c (Miltenyi Biotec Norden, Lund, Sweden), CD14 (DAKO, Copenhagen, Denmark), CD40 (BD Biosciences), CD80 (Immunotech, Marseilles, France), CD83 (BD Biosciences), CD86 (BD Biosciences). FlowJo™ software was used (ver. 7.2.4; Tree Star, Inc., Ashland, OR) for data analyses.

### Quantification of IL20RA, IL20RB, and IL22RA gene expression in MDDCs

Cells were lysed by adding 100 μl of cell lysis buffer, repeated pipetting and storing at -80 °C (Promega Biotech AB, Nacka, Sweden). Total RNA was purified with the SV Total RNA Isolation System™ (Promega) following the manufacturer’s instruction. Quantitative reverse transcriptase (qRT) PCR was performed on RNA extracted from the MDDCs of 6 healthy donors and using Taqman™ Reverse Transcription Reagents with random hexameric oligonucleotides following the manufacturer’s instructions (Applied Biosystems, Waltham, MA). The samples with complementary DNA (cDNA) were investigated by PCR using IL20RA, IL20RB, IL22R, and GAPDH primers (Applied Biosystems). The gene expression was quantified by the relative standard curve method [19].

### Interleukin-induced STAT, ERK, JNK, and p38 MAPK phosphorylation

Phosphorylation of signaling molecules in MDDCs treated with IL-20, IL-10 (cat.no. 200-10; Perprotech EC, London, UK), IL-19 (1035-IL/CF; R&D systems, Minneapolis, MN) or IL-24 (1965-IL/CF; R&D systems) at concentrations of 0.001, 0.01, 0.1, 1, or 10 ng/ml for 10 or 30 min before harvest (Fig. 1b) was analyzed by commercially available multiplex assays. The 3-plex assay measured the phosphorylation level of STAT1 (of residue of Tyr-701), STAT3, and STAT5a⁄b with antibody coupled beads (LHO0005; Invitrogen, Waltham, MA). The 4-plex assay (171-304004; Bio-Rad Laboratories, Inc., Hercules, CA) determined the phosphorylation level of p38 MAPK (of residue Thr-180 & Tyr- 182) as well as ERK1/ERK2, JNK, and STAT6 also by antibody-coupled beads.

### Assays for measuring the influence of interleukins on MDDCs migration

Migration assays were performed with the basement membrane extract (BME) Cell Invasion Assay™ (Trevigen, Gaithersburg, MD). Membranes with a pore size of 8 μm separating the upper and lower chambers in modified Boyden chambers were coated with BME according to the manufacturer’s instructions. MDDCs, either simulated with LPS or cytokines or without stimulations, (Fig. 1b) were then added to upper chambers of the modified Boyden chambers over the layer of coagulated BME. RPMI-1640/Gln with 10 % (v/v) human AB serum was added to the lower chamber as chemoattractant. Following one hour of incubation, migrated cells were released from the lower side of the membrane and stained with fluorescent dye was used to enable quantification in the Victor™ X3 multi label plate reader (PerkinElmer, Waltham, MA).

### Light microscopy and assays for CD18 shedding

MDDCs were cultured in Lab-Tek 2™ chamber slides (NUNC) with a quartz bottom. Imaging was made with a Leica (Wetzlar, Germany) inverted microscope equipped with an Automatic Thermocontrol System™ kept at 37 °C. For each slide a total of 10 images were made and combined by “z stacking” (Leica Application Suite). Detection of soluble (s)CD18 (Fig. 1b) in a time-resolved immunofluorometric assay (TRIFMA) in MDDC culture supernatants was carried out as described earlier [13, 20].

### Statistical methods and calculations on experimental data

Expression of cell surface markers were analyzed by flow cytometry and compared using paired two-tailed Student’s t-tests with Bonferroni’s corrections of the level of significance. Findings were considered significant if *p* <0.05/*N*, where *N* is the number of comparisons. A similar analysis was applied for data for the phosphorylation of STAT3 and p38 as well as data on expression of the IL-20 receptors.

The migration of MDDCs through BME layer, in response to interleukin treatment, was compared based on the number of migrated cells (MC) essentially reflecting the rate of migration since the BME layer thickness and number of applied cells were kept constant for the compared experiments. The mean value was obtained from triplicate measurements and reported as the harmonic mean, i.e.,_._

1 $$ \overline{MC}{\left(\frac{1}{3}{\displaystyle \sum_{i=1}^3\frac{1}{M{C}_i}}\right)}^{-1} $$

Variance homogeneity (Bartlett’s test) was obtained by inverse transformation of the number of migrated cells $\left(1/\overline{MC}\right)$. The different levels of IL-20 concentrations were compared by ANOVA with repeated measurements of 1/*MC*. A dose-response relationship between IL-20 concentration and MC was investigated based on a quadratic polynomial with

2 $$ 1/\overline{MC}=a\times {\left({ \log}_{10}{\uprho}_{IL-20}\right)}^2 + b\times { \log}_{10}{\uprho}_{IL-20}+\mathrm{c}, $$

where ρIL-20 is the mass concentration of IL-20 (in g/l) and *a*, *b*, and *c* are the polynomial constants determined from fitting Eq. 2 to the experimental data. In this model, the dose-response relationship between the IL-20 concentration and the harmonic mean of MC permit both positive and negative effects of IL-20 on *MC*. The data were tested against the hypothesis of no overall effect of IL-20, *i.e.*, *a* = *b* = 0, reducing Equation 2 to $1/\overline{MC}=\mathrm{c}$. For each MDDCs culture, the shedding of CD18 was analyzed by TRIFMA in three dilutions of the culture supernatant in duplicate wells. The influence of IL-20 treatment was tested by an ANOVA with the degrees of freedom corrected for the repeated measurements made for each culture.

## Results and discussion

### Effects of LPS and IL-20 on maturation of MDDCs

Several cytokines are known to play a role in the maturation of DC by enhancing the cell surface expression of proteins central to the function of DC, notably those involved in Ag presentation, signaling, and co-stimulation. To permit a study of such effects, we established a procedure (Fig. 1) for differentiating in vitro human monocytes into DC-like cells with a cell surface marker expression resembling immature DC in the skin. Following 6 days of culture, these cells showed an excellent viability (97–99 %). The maturation state of the MDDC was investigated by flow cytometry comparing the cell surface expression of maturation markers with or without LPS stimulation, which is a well-characterized agent for maturating DC. The panel of markers reflecting the Ag-presentation capability was chosen to include MHC class II molecules (HLA-DR shown in Additional file 1: Figure S1A) and the MHC-like molecules CD1a and CD1c (Additional file 1: Figure S1B,C). The signaling molecule CD40 (Additional file 1: Figure S1D), the T-cell stimulatory and regulating molecule CD83, and the costimulatory molecules CD80 and CD86 (Additional file 1: Figure S1E-G) are important in the signaling between DC and T lymphocytes [21]. CD14 is a pattern recognition molecules involved in the recognition of LPS and thus contributes to functions involved in the early events of immune recognition in case of microbial challenge [22].

**Fig. 2:**
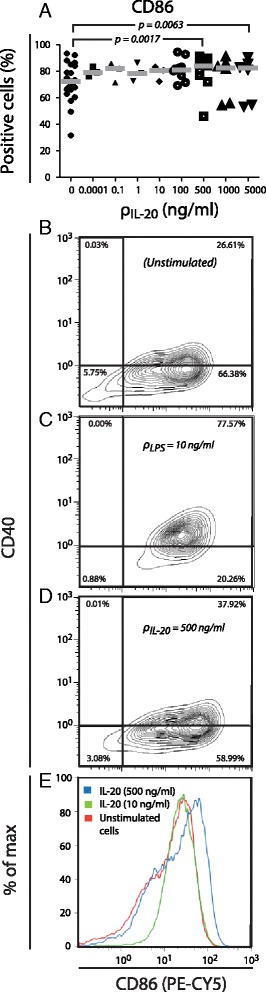
CD86 expression of IL-20-treated MDDCs. Cells were treated according to Incubation III outlined in Fig. 1b with addition of IL-20 to the culture 4-6 h before harvest. IL-20 was applied in concentrations (ρIL-20) of 0.001-5000 ng/ml and cells from 3-11 donors were analyzed. Results were based on MDDCs from Donors #1-25. **a** The percentage of CD86 positive MDDCs following incubation without or with IL-20 in the concentration stated on the abscissa. Bars indicate the statistical significant difference between treated and non-treated MDDCs cultures as analyzed by a t-test (**b**-**d**). Flow cytometric determination of the level of staining with antibodies to CD40 and CD86. As a representative case, results from the analysis of Donor 1 is shown, either as unstimulated (**b**), stimulated with LPS (**c**), or stimulated with IL-20 (**d**). **e** Histogram showing the distribution of staining intensities with Ab to CD86, either for untreated culture or for cultures treated with IL-20

The MDDCs were used to investigate the potential role of IL-20 in maturation of DCs (Fig. 2). Treatment of these cells with 500 or 5000 ng/ml of IL-20 for 4–6 h, significantly increased (*p* = 0.0017 and 0.0063, respectively) the percentage of CD86-posive cells (Fig. 2a). Interestingly, in a paired comparison with untreated cultures, the largest increment in CD86 positive cells following IL-20 treatment was observed in MDDCs cultures with relatively few CD86 positive cells before treatment, i.e. in the most immature MDDCs (data not shown). Furthermore, compared to untreated cells (Fig. 2b), only a subset of MDDCs responded to the treatment with 500 ng/ml IL-20 (Fig. 2d,e) and reached a higher CD86 expression level, surpassing the CD86 level following the treatment with LPS (Fig. 2c-e). Unlike the maturation induced by LPS (Additional file 1: Figure S1), no changes in the expression level of CD40 or the percentage of MDDCs positive for CD40 (Fig. 2d), HLA-DR, CD80, or CD83 (data not shown) was detected following stimulation with IL-20.

### IL-20 receptor expression, interleukin binding, and signaling

**Fig. 3:**
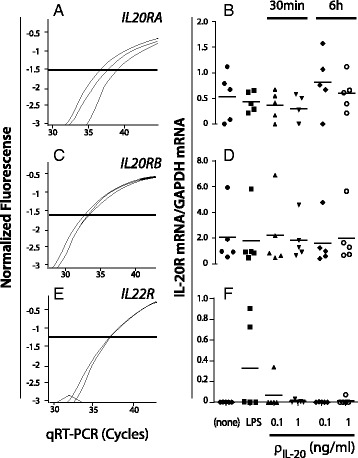
IL-20 receptor mRNA expression in LPS and IL-20-treated MDDCs. MDDCs were treated with LPS or IL-20, followed by isolation and quantification of mRNA transcripts from the *IL20RA* (**a**,**b**), *IL20RB* (**c**,**d**), or *IL22R* (**e**,**f**) genes by qRT-PCR. For each analysis, cells from six donors were used with the IL-20 receptor gene transcription normalization to the *GAPDH* mRNA transcripts. Results were based on MDDCs from Donors #25-30

The MDDC transcription of receptors for IL-20 was analyzed by quantitative RT-PCR (qRT-PCR) with the signals normalized to transcription from the *GAPDH* gene. Unstimulated MDDCs made transcripts of both *IL20RA* and *IL20RB* genes (Fig. 3a-d) suggesting that required proteins were expressed to ensure a functional IL-20 receptor complex. The IL20R mRNA transcription was not affected by treating the MDDCs with LPS nor IL-20 in concentrations of 0.1 or 1 ng/ml and incubation times varying from 30 min to 6 h (Fig. 3b,d). To explore other sources of IL-20 receptors the transcription from the *IL22R* locus was also analyzed. *IL22R* mRNA was not expressed in untreated MDDCs (Fig. 3e,f). In two of six donors a treatment with 10 ng/ml LPS for 24 h induced transcription of *IL22R* mRNA (Fig. 3f). The LPS-induced *IL22R* mRNA transcription was 0.3 relative to *GAPDH*.

**Fig. 4:**
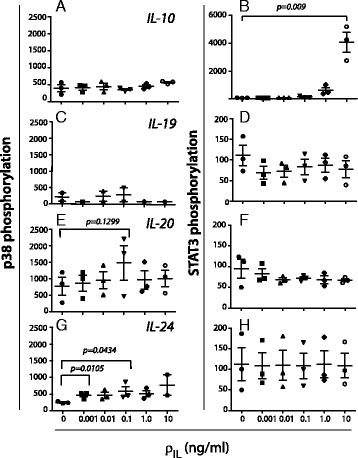
p38 MAPK and STAT3 phosphorylation in MDDCs pretreated by cytokines from the IL-10 family. Phosphorylation of p38 was measured for Thr-180 and Tyr-182 and for Tyr-705 in STAT3. Data were based on cellular cultures derived from 3 donors and measured in duplicates. The data are shown as the mean values of (*arbitrary*) fluorescence units with error bars indicating the SD. Interleukin pretreatment was started 10 min before harvest with the cytokines added at concentrations (ρ_IL_) of 0.001, 0.1, 1, or 10 ng/ml (Incubation V in Fig. 1b) and compared with cultures without cytokine addition. The influence was tested of IL-10 on p38 (**a**) and STAT3 (**b**) phosphorylation, and similarly for IL-19 (**c**,**d**), IL-20 (**e**,**f**), and IL-24 (**g**,**h**)

To investigate the functional relevance of the IL20R expression, we analyzed the response to IL-10 family interleukins, which share the property of binding to both of these receptor complexes. By comparing the outcome considering phosphorylation of p38 MAPK and STAT3 substrates, we collected more information on the signaling pathway usage by these interleukins (Fig. 4). MDDCs were incubated with IL-10, a known potent activator of STAT3 in MDDCs [23]. As expected, IL-10 had no influence on phosphorylation of the p38 (Fig. 4a) while pretreatment with 10 ng/ml IL-10 induced a 4,000-fold increase in the STAT3 phosphorylation (*p* = 0.009) compared with untreated MDDCs (Fig. 4b). IL-19 appeared neither to influence the p38 nor the STAT3 phosphorylation (Fig. 4c,d). By contrast, both IL-20 (Fig. 4e) and IL-24 (Fig. 4g) increased the p38 phosphorylation, but these interleukins did not significantly alter the STAT3 phosphorylation (Fig. 4f,h). The p38 phosphorylation was altered following the treatment with of 0.1 ng/ml of both cytokines individually administered (Fig. 4e,g). Interestingly, the IL-20 induced p38 phosphorylation peaked at 0.1 ng/ml, while lower or higher concentrations produced lower mean values among the tested cell cultures (Fig. 4e). In two of three donors, the p38 phosphorylation level was more than doubled when MDDCs were pretreated with 0.1 ng/ml of IL-20. However, the increase in p38 phosphorylation level was not statistically significant when analyzed with a paired t-test (*p* = 0.1299). Following the pre-treatment of MDDCs with IL-24, the p38 phosphorylation level was raised in all samples and for all IL-24 concentrations. For samples pretreated with IL-24 at concentrations of 0.001 and 0.1ng/ml, the increase in the mean phosphorylation level was statistically significant (*p* = 0.0105 and 0.0434, respectively) (Fig. 4g). IL-19, IL-20 and IL-24 did not affect the phosphorylation levels of ERK-1/2, JNK, STAT1, STAT5, or STAT6 (data not shown).

### Functional influences of IL-20 on migration and adhesion receptor shedding in MDDCs

To investigate the functional consequences of IL-20 mediated signaling in MDDCs, an analysis linking IL-20 to known properties of DCs or LC in skin (notably in psoriatic skin lesions) was required. Several lines of evidence suggested the importance of these cells in transporting the Ag into draining lymph nodes. Additionally, the report by Cumberbatch et al*.* [9], suggested an aberrant trafficking of these cells in psoriatic skin lesions.

For in vitro analysis of MDDC migration we used a modified Boyden chamber assay with fluorescence labeling of the MDDCs. The influence of IL-19, IL-20, and IL-24 was probed by pre-incubation of the cells with the interleukins before applying the cells into the upper chamber where an ECM-like matrix covered a porous membrane. To stimulate migration, human AB serum was added as a chemoattractant in the lower chamber.

**Fig. 5:**
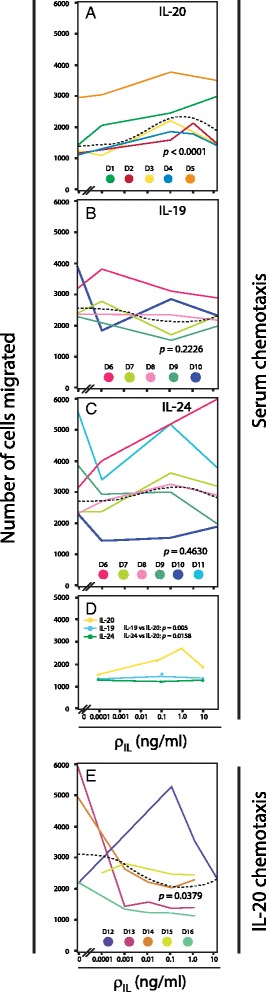
Influence of IL-10 family interleukins on MDDCs migration. **a**-**d** The migration of MDDCs towards an AB human serum gradient through an ECM-like coagulate on a membrane with 8-μm pores in a modified Boyden chamber. The MDDCs were incubated with or without addition of IL-19, IL-20, or IL-24 at concentrations (ρIL) of 0.0001, 0.001, 0.01, 0.1, 1.0, or 10 ng/ml for 30 min. **a** Migration of IL-20-pretreated MDDCs derived. **b** Migration of IL-19 pretreated-MDDCs. **c** Migration of IL-24 pretreated-MDDCs. **d** Summary of the dose-response curves. **e** Analysis of the role of IL-20 as a chemoattractant for MDDCs where IL-20 applied in the lower chamber (instead of AB serum)

Pre-incubations with interleukins were carried over a broad range from 0–10 ng/ml (Fig. 5). In four of five donors, a peak in the number of migrated cells was observed following pre-incubations with 0.1–1.0 ng/ml of IL-20 (Fig. 5a). However, to obtain a robust statistical analysis of the collected set of data, we used the quadratic polynomial described by Eq. 2 in order to establish a connection between the interleukin concentration and MDDCs migration. This approach returned a statistical significant (*p* < 0.0001) model with a biphasic migration response to pre-incubation with IL-20 (Fig. 5a). The constants *a*, *b* and *c* in Eq. 2 were estimated 0.045 ± 0.011, -0.046 ± 0.013, and 0.439 ± 0.091, respectively (indicated as the estimated value ± standard error). The coefficient for the quadratic part of Eq. 2 (*a*) was highly significant (*p* = 0.0004). This implies that the quadratic part cannot be ignored in the curve fitting and thus suggesting a biphasic response to the treatment with IL-20. By contrast, pre-incubation with IL-19 or IL-24 did not support any significant statistical connection between the applied interleukin concentration and migration (Fig. 5b,c). To further explore this point, we also calculated the expected mean response to pre-incubation with IL-20, IL-19 and IL-24 (Fig. 5a-c). This comparison showed that that IL-20 was able to induce a migration approximately 2-fold higher than the base line or in the MDDCs cultures treated with IL-19 and IL-24 (Fig. 5d).

We also tested whether IL-20 acted as a chemoattractant for MDDCs (Fig. 5e). In this case, IL-20 was added to the lower part of the Boyden chamber. The migration of MDDCs from four of the five donors showed no ability of IL-20 to attract the MDDCs. By contrast, addition of IL-20 to the lower chamber reduced the MDDCs migration (Fig. 5e). This finding was supported by the statistical significance of the curve fitting (*p* = 0.0379) according to Eq. 2. The maximal effect was observed following treatment with 0.1 and 1 ng/ml of IL-20. This influence of IL-20 was clearly titratable and vanished at concentrations lower than 0.01-0.01 ng/ml. While Eq. 2 was able to account for the data, it should be noted that an even simpler approach using a simple linear relationship between the IL-20 concentrations produced a stronger significance (*p* = 0.0226).

Cell migration, in particular that mediated by integrins, is closely linked with mechanisms supporting adhesion. Recent studies shown that β2 (CD18) integrins are shed from the leukocyte cell surface in inflammatory diseases and under certain experimental conditions in vitro [13, 15]. We employed assays described earlier [13] to analyze the connection between IL-20 and CD18 integrin shedding.

**Fig. 6:**
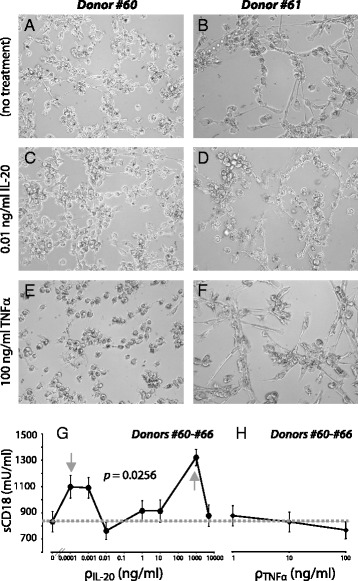
Adhesive properties of cytokine-treated MDDCs. Cells were incubated with IL-20 or TNFα prior to microscope imaging (**a**-**f**) or analysis of β2 (CD18) integrin adhesion molecules shedding (**g**,**h**). MDDCs were imaged either as untreated (**a**,**b**), following treatment with 0.01 ng/ml IL-20 (**c**,**d**), or with 100 ng/ml TNFα (**e**,**f**). **g**,**h** CD18 shedding from MDDCs treated with cytokines. Based on cells from seven donors (#60-#66), culture supernatants from cytokine-treated MDDCs were applied in analyses of the concentration of sCD18. Comparison of sCD18 concentration (in mU/ml) obtained from either untreated cultures, cultures stimulated with 0.01-5000 ng/ml of IL-20 (**g**), or 1-100 ng/ml of TNFα (**h**)

A simple way of monitoring alterations in cellular adhesion involves microscopy of surface-bound cells [24]. During the culture of MDDCs we observed that IL-20-exposed cultures showed subtle, yet persistent, changes in overall morphology and cellular distribution compared with untreated controls. In Fig. 6 such changes are shown for cells derived from Donor #61 (Fig. 6a,c) and #62 (8B,D). The observation of that IL-20 treated cells (Fig. 6c,d) were more likely to form clusters than non-treated cells (Fig. 6a,b) suggests that intercellular adhesion was changed. However, as demonstrated by treatment with TNFα (Fig. 6e,f), the donor variations were large and the types of morphological changes were difficult to characterize by uniform parameters.

Soluble CD18 levels in the supernatants were measured to quantify the processes relevant to cellular adhesion in MDDCs derived from seven donors, following seven days of culture with addition of cytokines on Day 6 (Fig. 6g,h) [13]. IL-20 was applied in concentrations ranging 0.0001 to 5000 ng/ml. In Fig. 6g, the sCD18 concentration was plotted as a function of the IL-20 concentration. Testing with ANOVA whether the mean value for sCD18 in each group of IL-20 treatments were equal was rejected with *p* = 0.0256 (Fig. 6g). In the interval between 0.0001 and 1000 ng/ml (marked by grey arrows), the profile of IL-20-influenced shedding showed a tendency towards a biphasic response, where the lowest shedding occurred for treatment with 0.01–10 ng/ml relative to either lower or higher dosages (Fig. 6g). Previously, we observed that CD18 shedding requires viable cells [13], and hence we suspected this biphasic pattern could be due to cell death. However, analyses with flow cytometry did not support this explanation (data not shown). TNFα was previously reported to enhance CD18 shedding in cultures of human mononuclear leukocytes and granulocytes [13]. However, application of this cytokine to the cultures of MDDCs moderately reduced the shedding (Fig. 6h). In this case, visual inspection of the cellular cultures indicated some loss of viable cells in the wells (Fig. 6e-f).

Importantly, Gjelstrup et al*.* [13] reported the finding of large oligomeric species of CD18 in human plasma and in recombinant soluble CD11a/CD18. Crystallographic data [25] suggested that the oligomers contained from two chains (1 × CD11a/CD18) up to at least a total of eight chains (4 × CD11a/CD18). However, it has never been tested whether CD18 oligomers are formed in cellular cultures and what conditions may affect the oligomeric size.

**Fig. 7:**
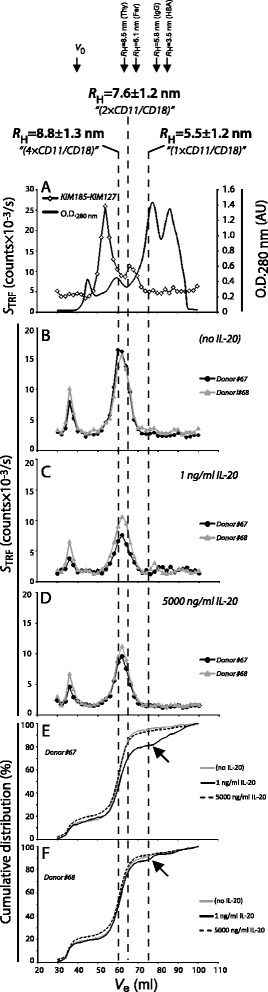
Distribution of sCD11/CD18 oligomers in MDDCs culture supernatants. **a** GPC followed by TRIFMA detection of sCD11/CD18 in a “sandwich-type” assay with the monoclonal Ab KIM185 to CD18 as a capture Ab and KIM127. The plot shows the TRIFMA signal (*S*_TRF_, read on the left ordinate) and the optical density (OD) at λ = 280 nm (read on the right ordinate) in the fractions plotted as a function of the elution volume, *V*_e_. The elution volumes of oligomeric recombinant soluble CD11a/CD18 (established in a previous study [13] using the same chromatography equipment) are indicated hatched lines together with the elution volumes of the hydrodynamic radius calibration markers thyroglobulin, ferritin, IgG, and albumin. **b**-**d** Distribution of sCD11/CD18 oligomers in cell culture supernatant from MDDCs. The analysis was carried out with culture supernatants derived from two Donors (#67 and #68), either left untreated or treated with 1 or 5000 ng/ml IL-20 (Incubation VII in Fig. 1b) The sCD11/CD18 signal in the fractions was measured with the KIM185-KIM127 assay and plotted as a function of *V*_e_. (**e**,**f**) Cumulative distribution of the CD18 signal in the GPC fractions. The cumulative distribution profile was calculated by plotting $100\%\ \frac{{\displaystyle {\sum}_{V_e=30 ml}^{V_e=i}{S}_{TRF}\left({V}_e\right)}}{{\displaystyle \sum {S}_{TRF}}}$ as a function of *V*_e_, where *S*_TRF_(*V*_e_) is the CD18 signal for the fraction with elution volume *V*_e_ and ∑*S*_*TRF*_ the sum of signals for all fractions eluting from 30 ml to 100 ml, both end points included. Black arrows indicate a feature in the distribution profile shared between the donors

Supernatant from two independent cultures of MDDC were analyzed by GPC (Fig. 7). For comparison, the CD18 oligomeric profile in human citrate plasma was also included (Fig. 7a). In both IL-20-treated (Fig. 7c,d) and untreated cultures (Fig. 7b), the CD18 oligomers were mainly distributed in two peaks eluting at the column void volume and in volumes corresponding to a hydrodynamic radius of ~8 nm. As indicated with hatched lines this corresponds to the size of complexes also found in plasma and in recombinant soluble CD11a/CD18. By contrast, the oligomers eluting in the void volume (Fig. 7b-d) had no corresponding oligomers in plasma (Fig. 7a). To study the influence of IL-20 on oligomer size, the raw data (Fig. 7b-c) were analyzed by plotting the cumulative distribution of CD18 signals in the fraction eluting with *V*_e_ from 34 to100 ml (Fig. 7e,f). In the supernatants from Donor #67, it was clear that treatment with 1 ng/ml of IL-20 reduced the abundance of oligomers with a *R*_H_ larger than ~ 7.5 nm compared with either no treatment or treatment with 5,000 ng/ml IL-20. By contrast, treatment with 5000 ng/ml appeared to induce a small increase in the abundance of these larger oligomers compared with either of the two other culture conditions. Qualitatively these difference were also found for Donor #68 (Fig. 7f), even with shared detailed features in the distribution profile (indicated with black arrows in Fig. 7e,f), albeit the quantitative difference was smaller for this donor.

## Conclusion

We established in vitro culture conditions that enabled a phenotypic expression of cell surface markers mimicking the immature DCs in humans, further confirmed by the vigorous response of these cells to LPS-induced maturation. Pretreatment of the MDDCs with IL-20 resulted in an increased CD86 expression. CD86 contributes to the formation of the immunological synapse between DCs and T-cells [21, 26, 27], which is critical in the development of psoriasis. Only high IL-20 concentrations (500-5000 ng/ml) resulted in a significant increase in the CD86 positive cells. As a comparison, the mean concentration of IL-20 in serum from patients suffering rheumatoid arthritis was reported to be 0.282 ng/ml [3]. However, the local environment of the psoriatic epidermis, which includes the IL-20 producing keratinocytes and MDDCs in close proximity, is likely to present IL-20 concentrations higher than the systemic concentration [28].

Previous studies found transcription of mRNA in MDDCs encoding the IL-20RB chain, while transcripts encoding IL-20RA or IL-22R were not reported [6] or only found in trace amounts [29]. By testing the immature MDDCs, transcripts encoding IL-20RA and IL-20RB were detected, albeit only weakly, suggesting the formation of functional receptor complexes in the membrane of MDDCs.

No previous reports addressed the IL-20-induced signal transduction in leukocytes. In MDDCs, IL-20 and IL-24 induced p38 phosphorylation in MDDCs, while IL-10 and IL-19 did not. IL-24 was the most potent stimulator of the p38 MAPK pathway, while IL-20 showed a weaker mean phosphorylation. For both IL-20 and IL-24 the most potent response was observed for treatment with 0.1 ng/ml interleukin.

Cumberbatch et al*.* reported that Langerhans cells are trapped in non-lesional psoriatic skin [9]. IL-20 is known to affect migration of neutrophils, chemotaxis of synovial fibroblasts chemotaxis in rheumatoid arthritis, and vascular tube-formation by endothelial cells [30, 31]. Our findings now suggest that IL-20 potentiates MDDCs migration towards a chemoattractant. IL-20 itself did not act as a chemoattractant but decreased migration in higher IL-20 concentrations. Interestingly, in our study a similar influence by other IL-10 interleukin family members, i.e., IL-19 and IL-24, was not found. A simple explanation, in agreement with the observations for the IL-20-mediated signaling in MDDCs, was offered recently by a structural characterization of the ternary complex between IL-20 and the IL20-A/IL-20B receptor. From these data as well as direct measurements of the binding of IL-20 and IL-19, it appeared that the IL-19 binds to the IL-20A/IL-20B receptor 10-fold weaker than IL-20 [32].

Based on the hypothesis that CD18 shedding is critical for the migration of myeloid cells [33, 34], we analyzed the influence of IL-20 on CD18 shedding in cultures of MDDCs. As in the case of the migrational influence of IL-20, no simple relation between the applied IL-20 concentration and CD18 shedding was found. However, there was an indication that IL-20 in the concentration interval 0.01-10 ng/ml induced lower shedding than either lower or higher concentrations. A more detailed investigation was carried out by analyzing the CD18 oligomer size distribution in cultures treated with IL-20 compared to untreated controls. The most striking effect was again observed for treatment with 1 ng/ml IL-20, which apparently was able to reduce the relative abundance of larger oligomers, albeit with marked differences in the quantitative response among the two independent cultures tested. Nevertheless, our data suggest that not only the level of shedding but also the CD18 oligomer size is variable influenced by cytokine stimulation of MDDC. This proposal seems entirely consistent with the multiple capacities of the complex proteolytic mechanisms responsible for regulating the shedding [16, 33, 35]. Interestingly, the observation that such changes occurs by treatment with 1 ng/ml IL-20, which was also found to affect migration suggest a potential link between the structural formation of CD18 oligomers and migration.

In conclusion, our findings suggest that IL-20 has an influence on processes central to the function of DCs. However, the responses found in our report and in the work by others were either weak or subject to considerable donor variation. This situation questions if IL-20 is a significant part of the normophysiological functions of DCs. Our findings may propose an aberrant influence of IL-20 in the case of the high concentrations as often found in psoriasis.

## Additional file

Additional file 1: Figure S1. LPS-induced maturation of MDDCs monitored by the expression of cell surface markers. For each maturation marker the expression was investigated in culture receiving LPS treatment (10 ng per ml of culture medium, indicated as Incubation II in Fig. 1b) or in control cultures receiving treatment with vehicle only (indicated as Incubation I in Fig. 1b). The percentage of marker-positive cells were established by flow cytometry staining for HLA-DR (A), CD1a (B), CD1c (C), CD40 (D), CD80 (E), CD83 (F), CD86 (G), and CD14 (H). Results were based on MDDCs from Donors #1-25. The p value (indicated in italics) was calculated using a two-tailed Student’s t-test. (PDF 282 kb)

## Abbreviations

BME: basement membrane extract
DC: dendritic cells
ECM: extracellular matrix
FS: forward scatter
GPC: gel permeation chromatography
HSA: human serum albumin
LC: langerhans cells
LPS: lipopolysaccharide
MDDC: monocyte-derived DC
qRT-PCR: quantitative reverse transcriptase-PCR
sCD18: soluble CD18
SS: side scatter
