# Supplementary figures and images for: Interleukin 20 regulates dendritic cell migration and expression of co-stimulatory molecules
Source: Mol Cell Ther. 2016 Jan 26;4:1. doi: 10.1186/s40591-016-0046-x (PMC4728801; doi:10.1186/s40591-016-0046-x)

Fig. S1; Bech *et al.*

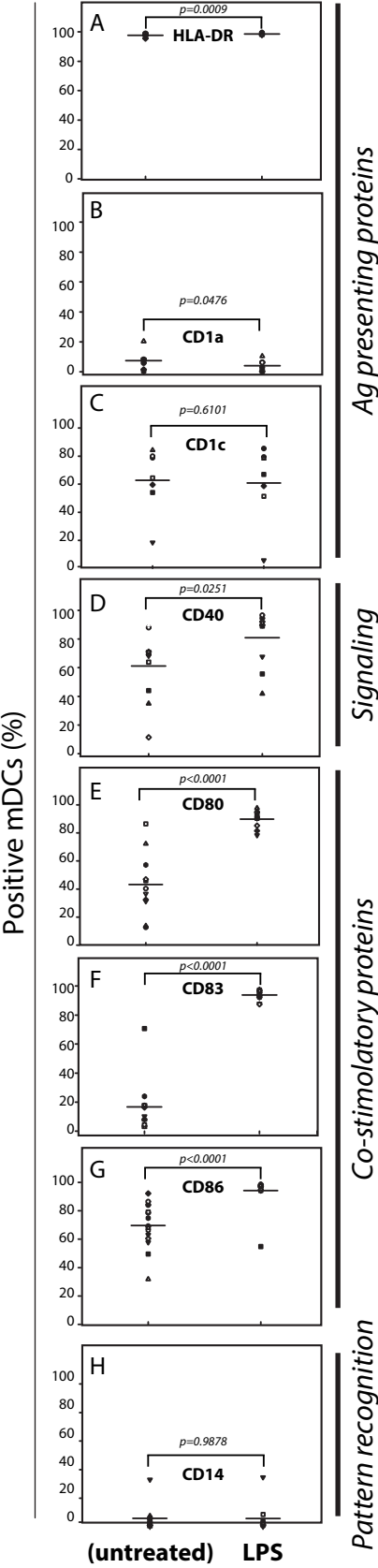

Supplement: Additional file 1: Figure S1. — LPS-induced maturation of MDDCs monitored by the expression of cell surface markers. For each maturation marker the expression was investigated in culture receiving LPS treatment (10 ng per ml of culture medium, indicated as Incubation II in Fig. 1b) or in control cultures receiving treatment with vehicle only (indicated as Incubation I in Fig. 1b). The percentage of marker-positive cells were established by flow cytometry staining for HLA-DR (A), CD1a (B), CD1c (C), CD40 (D), CD80 (E), CD83 (F), CD86 (G), and CD14 (H). Results were based on MDDCs from Donors #1-25. The p value (indicated in italics) was calculated using a two-tailed Student’s t-test. (PDF 282 kb) [file 40591_2016_46_MOESM1_ESM.pdf]
